# *FTO* rs62033406 A>G associated with the risk of osteonecrosis of the femoral head among the Chinese Han population

**DOI:** 10.1186/s12920-022-01283-z

**Published:** 2022-06-15

**Authors:** Yuan Wang, Wei Zhong, Shaofeng Wang, Yang Yang, Bing Zhu

**Affiliations:** Department of Joint Surgery, Affiliated Hospital of Weifang Medical University, # 2428 Yuhe Road, Weifang, 261031 Shandong China

**Keywords:** Osteonecrosis of the femoral head, *FTO* polymorphisms, Risk factor, MDR analysis

## Abstract

**Background:**

*Fat mass and obesity-related (FTO)* mRNA was downregulated in osteonecrosis patients. The study aimed to evaluate the correlation between *FTO* polymorphisms and the susceptibility of osteonecrosis of the femoral head (ONFH).

**Methods:**

Six polymorphisms in *FTO* were genotyped via the Agena MassARRAY in 498 ONFH patients and 498 healthy controls. Multiple genetic models were used to assess the correlation between *FTO* polymorphisms and ONFH risk by SNPStats. Odds ratios (ORs) and 95% confidence intervals (CIs) were estimated using a logistic regression model adjusted by age, gender, smoking and drinking.

**Results:**

The risk-increasing association of rs62033406 A>G with ONFH was found (OR = 1.25, 95% CI 1.05–1.50, *p* = 0.014). Specially, *FTO* rs62033406 A>G was related to the risk of ONFH in the subgroup at age > 51 years (OR = 1.25, *p* = 4.00 × 10^–4^), females (OR = 1.74, *p* = 1.00 × 10^–4^), smokers (OR = 1.82, *p* = 0.005) and drinkers (OR = 1.89, *p* = 0.002), respectively. The best multi–loci model was the five–loci model, a combination of rs9930333 T>G, rs1558902 T>A, rs56094641 A>G, rs3751812 G>T, and rs62033406 A>G (testing accuracy, 0.5351; *p* = 0.0004; cross–validation consistency, 10/10).

**Conclusion:**

Our study first revealed that *FTO* rs62033406 A>G was a risk factor for ONFH among the Chinese Han population, which might provide the new candidate gene for elucidating the pathogenesis of ONFH.

**Supplementary Information:**

The online version contains supplementary material available at 10.1186/s12920-022-01283-z.

## Introduction

Osteonecrosis of the femoral head (ONFH), also defined as avascular necrosis of the femoral head, is characterized by bone cell death caused by venous stasis of the femoral head or impaired or interrupted arterial blood supply [1]. The onset of ONFH remains increasing every year around the world. It is estimated that there are 8.12 million adults with ONFH in China alone [2]. The average age of the affected patients is 50.4 years and there is a male to female ratio of 2.1:1 [3]. ONFH is a multifactorial disease caused by a complex interplay of genetic and environmental factors. The use of corticosteroids, alcohol overconsumption, smoking, high blood lipid level, and obesity are all high-risk factors for ONFH [4]. In recent studies, emerging lines of evidence have suggested genetic factors and hereditary forms play a pivotal role in ONFH development [5]. For example, *NOS3*, *IL-1B*, *ABCB1* and *CYP450* single nucleotide polymorphisms (SNPs) were closely related to the susceptibility of ONFH [6–8]. However, many variants contributed to ONFH remain to be identified.

The fat mass and obesity-related (*FTO*) gene has been reported to be involved in common obesity and body mass index [9]. Deletion of *FTO* in mice might lead to increased death of osteoblasts and bone loss, suggesting that the function of *FTO* was to maintain bone mass and to protect osteoblasts from genotoxic damage [10]. Recent study indicated that overexpression of *FTO* induces osteogenic differentiation of C3H10T1/2 cells [11]. Pervious findings demonstrated that GDF11-*FTO*- PPARγ axis inhibited bone formation and promoted the transformation of osteoporotic bone marrow mesenchymal stem cells (BMSCs) to adipocytes during osteoporosis [12]. These studies indicated that *FTO* played an important role in the death and differentiation of osteoblasts. *FTO* mRNA was downregulated in osteoporosis patients and osteonecrosis patients [13]. *FTO* polymorphisms have been investigated in many bone-related diseases, such as hip fracture, osteoporosis and osteoarthritis [14–16], but not in ONFH. Therefore, research on the possible association between *FTO* gene and ONFH may be particularly interesting due to its potential biological significance.

Here, six SNPs (rs9930333 T>G, rs11642015 C>T, rs1558902 T>A, rs56094641 A>G, rs3751812 G>T, and rs62033406 A>G) in *FTO* were randomly selected for genotyping to assess the contribution of these SNPs to ONFH risk in the Chinese Han population, which might contribute to knowing about the role of *FTO* in the development of ONFH. Stratified analysis by age, sex, smoking, and drinking were used to explore the different association graph patterns in each subgroup, which might reflect the potential differences in their pathophysiology and ONFH risk factors.

## Materials and methods

### Study participants

In this study, we recruited 498 patients with ONFH and 498 healthy controls from the Affiliated Hospital of Weifang Medical University and Second Affiliated Hospital of Inner Mongolia Medical University from April 2015 to June 2021. All subjects were genetically unrelated Chinese Han population. Patients with ONFH had common clinical manifestations of hip pain, joint dysfunction, and lower limb muscular atrophy. ONFH was diagnosed by an X-ray examination, bone scan analyses and additional magnetic resonance imaging. Diagnostic criteria for imaging include the collapse of the femoral head, increased subchondral bone density, hip joint narrowness, and trabecular and marrow necrosis on histology. The inclusion criteria for health controls were defined as following criteria: no symptoms of hip disease, no tumor, no any lesions of anteroposterior and frog-leg lateral pelvic radiographs, no severe chronic diseases, and no history of thromboembolic disease. Demographic characteristics (age, gender, smoking, and drinking) and clinical data were obtained via a structured questionnaire and medical records, respectably. The identification of smokers and drinkers was based on the subjects’ self-report of whether they smoked or drank alcohol. For smoking, participants were classified as non-smokers (never) or smokers (including former or current smokers). Subjects who smoked one cigarette per day were classified as current smokers. For alcohol consumption, participants were classified as non-drinkers (never) or drinkers (including former or current alcohol drinkers). Subjects who drank at least 100 g of alcohol a week were considered drinkers. The study protocol was approved by the institutional review boards of Affiliated Hospital of Weifang Medical University, and was conducted in accordance with the ethical standards of the Declaration of Helsinki. Written informed consents were obtained from all participants.

### SNP selection and genotyping

Subsequently, 5 mL of peripheral blood from participants were collected into ethylene diamine tetraacetie acid (EDTA) tubes. Genomic DNA was isolated using genomic DNA purification Kit (GoldMag Co. Ltd. Xi’an City, China) and stored at − 80 °C until further analysis. The concentration and purity of DNA was measured by a Nanodrop 2000 (Thermo Scientific, Waltham, MA, USA).

We obtained the physical position of the *FTO* gene on the chromosome 16:53,701,692–54,158,512 and downloaded the ped file and info file for *FTO* variations in the CHB and CHS population through the e!GRCh37 (http://asia.ensembl.org/Homo_sapiens/Info/Index) database. Using Haploview software, we selected SNPs based on HWE > 0.01, MAF > 0.05, and Min Genotype > 75%. We further combined MassARRAY primer design software, HWE > 0.05, MAF > 0.05 and the call rate > 95% in our study population. Among the remaining SNPs, six candidate SNPs (rs9930333 T>G, rs11642015 C>T, rs1558902 T>A, rs56094641 A>G, rs3751812 G>T, and rs62033406 A>G) in *FTO* were randomly selected in order to study their potential role in ONFH risk. The potential functions of these polymorphisms were evaluated through the HaploReg v4.1 database (https://pubs.broadinstitute.org/mammals/haploreg/haploreg.php) and RegulomeDB database (https://regulome.stanford.edu/regulome-search/).

Agena MassARRAY system (Agena, San Diego, CA, USA) was used for SNPs genotyping according to the manufacturer instructions. The design of genotyping primers (Additional file 1: Table S1) and data management was carried out with incorporated software. The randomly selected samples (about 10%) were re-genotyped to control quality, and the reproducibility was 100%.

### Statistical analysis

The student t test for continuous variables and chi-square test for categorical variables were used to compare the differences between cases and controls, respectively. The genotype frequency of each SNP in the control and the case subjects were conformed to HWE by the χ^2^ test. Multiple genetic models were used to assess the contribution of *FTO* polymorphisms to the risk of ONFH by SNPStats (https://www.snpstats.net/start.htm). Odds ratios (ORs) and 95% confidence intervals (CIs) were estimated using a logistic regression model adjusted by age, gender, smoking and drinking. Further stratification analysis was performed to explore the impact of *FTO* SNPs on ONFH based on age, gender, smoking, drinking, and clinical stages. The noteworthy of the significant associations were assessed by false-positive report probability (FPRP) analysis, with 0.2 as a FPRP threshold and a prior probability of 0.1 [17, 18]. The SNP–SNP interactions in ONFH susceptibility was evaluated by multifactor dimensionality reduction (MDR) (version 3.0.2). Data analyses were analyzed by the SPSS 18.0 (SPSS, Chicago, IL), and a two‐tailed *p* < 0.05 was the threshold for statistical significance, whereas a value of corrected *p* < 0.05/6 was considered significant after Bonferroni correction.

## Results

### Characteristics of the participants

A total of 996 subjects (498 ONFH patients and 498 healthy controls) were included. The demographics and clinical information of subjects were shown in Table 1. The mean age of cases and controls were 51.77 ± 14.55 years and 50.38 ± 14.52 years, respectively. ONFH cases and controls were matched in age (*p* = 0.132), but there was a difference in sex (*p* = 0.046). The distribution of smoking, red blood cell (RBC), white blood cell (WBC), hemoglobin, platelet, leukocyte, monocytes, urea, and creatinine between the two groups were different (*p* < 0.05).

**Table 1:** Characteristics of patients with ONFH and controls

Variable	Cases	Control	*p*
N	498	498	
Age (year, mean ± SD)	51.77 ± 14.55	50.38 ± 14.52	0.132
> 51	273 (54.8%)	236 (47.4%)	
≤ 51	225 (45.2%)	262 (52.6%)	
Gender			
Males	280 (56.2%)	311 (62.4%)	**0.046**
Females	218 (43.8%)	187 (37.6%)	
Smoking			
Yes	219 (44.0%)	269 (54.0%)	**0.002**
No	279 (56.0%)	229 (46.0%)	
Alcohol consumption			
Yes	265 (53.2%)	256 (51.4%)	0.568
No	233 (46.8%)	242 (48.6%)	
Stage			
III/IV	192 (38.6%)		
I/II	67 (13.5%)		
Missing	235 (47.2%)		
RBC (10^9^/L)	3.91 ± 0.83	4.90 ± 0.48	** < 0.001**
WBC (10^9^/L)	7.67 ± 2.53	5.94 ± 1.59	** < 0.001**
Hemoglobin (g/L)	118.35 ± 20.11	149.76 ± 17.03	** < 0.001**
Platelet (10^9^/L)	197.74 ± 72.79	222.23 ± 58.94	** < 0.001**
Leukocyte (10^9^/L)	1.60 ± 0.65	1.87 ± 0.59	** < 0.001**
Monocytes (10^9^/L)	0.55 ± 0.26	0.42 ± 0.16	** < 0.001**
Serum uric acid (μmol/L)	266.67 ± 88.17	322.64 ± 80.7	0.337
Urea (μmol/L)	5.29 ± 5.35	5.06 ± 1.27	** < 0.001**
Creatinine (μmol/L)	57.32 ± 16.53	67.65 ± 12.57	** < 0.001**

ONFH, osteonecrosis of the femoral head; RBC, red blood cell; WBC, white blood cell

*p* values were calculated by χ^2^ test or Student’s t test

Bold indicate that *p* < 0.05 indicates statistical significance

### Association between FTO SNPs and the risk of ONFH

Six SNPs (rs9930333 T > G, rs11642015 C > T, rs1558902 T > A, rs56094641 A > G, rs3751812 G > T, and rs62033406 A > G) in *FTO* were genotyped, and the basic information of all SNPs was listed in Table 2. All selected polymorphisms were in HWE (*p* > 0.05), and the call rate was > 99.5%. The potential function of these polymorphisms by HaploReg v4.1 and RegulomeDB databases was displayed in Table 2.

**Table 2:** The information about *FTO* SNPs and the association with ONFH susceptibility in allele model

SNPs ID	Chr: position	Alleles (minor/major)	Frequency (MAF)		Function	*P *value for HWE		Call rate	OR (95% CI)	*P*	Haploreg	Regulome DB
			Case	Control		Control	Case					
rs9930333	16:53,766,065	G/T	0.154	0.157	Intronic	0.610	0.105	99.8%	0.98 (0.77–1.25)	0.853	Enhancer histone marks, Motifs changed	TF binding or DNase peak
rs11642015	16:53,768,582	T/C	0.109	0.107	Intronic	0.817	0.401	100.0%	1.02 (0.77–1.35)	0.885	Promoter histone marks, Enhancer histone marks, DNAse, Proteins bound, Motifs changed	TF binding + DNase peak
rs1558902	16:53,769,662	A/T	0.105	0.106	Intronic	0.814	0.524	100.0%	0.99 (0.74–1.32)	0.942	Promoter histone marks, Enhancer histone marks, DNAse, Motifs changed	TF binding or DNase peak
rs56094641	16:53,772,541	G/A	0.110	0.108	Intronic	0.999	0.427	100.0%	1.02 (0.77–1.35)	0.886	Promoter histone marks, Enhancer histone marks, DNAse, Motifs changed	Motif hit
rs3751812	16:53,784,548	T/G	0.107	0.108	Intronic	0.999	0.561	99.8%	0.98 (0.74–1.31)	0.910	Enhancer histone marks, DNAse, Motifs changed	TF binding + any motif + DNase peak
rs62033406	16:53,790,314	G/A	0.483	0.434	Intronic	0.465	0.215	99.9%	**1.22 (1.02–1.46)**	**0.028**	Enhancer histone marks, Motifs changed	Motif hit

Data from Haploreg (https://pubs.broadinstitute.org/mammals/haploreg/haploreg.php), and Regulome DB (https://regulome.stanford.edu/regulome-search/)

ONFH, osteonecrosis of the femoral head; SNP, single nucleotide polymorphism; IS, ischemic stroke; MAF, minor allele frequency; HWE, Hardy–Weinberg equilibrium

Bold indicate that *p* < 0.05 indicates statistical significance

In the allelic model, rs62033406-G was related to an increased risk of ONFH (OR = 1.22, 95% CI 1.02–1.46, *p* = 0.028). The other SNPs did not appear to be related to the susceptibility to ONFH. Genotypic model analysis was used to assess the associations of *FTO* SNPs with ONFH risk (Table 3). The risk-increasing association of rs62033406-G with ONFH was found in the codominant (OR = 1.54, 95% CI 1.14–2.07; OR = 1.52, 95% CI 1.06–2.18; *p* = 0.011), dominant (OR = 1.53, 95% CI 1.16–2.03, *p* = 0.003), and log-additive (OR = 1.25, 95% CI 1.05–1.50, *p* = 0.014) models. The significance of the dominant model still existed after Bonferroni correction.

**Table 3:** Association between *FTO* polymorphisms and ONFH risk

SNP ID	Model	Genotype	Case	Control	OR (95% CI)	*P* value	AIC	BIC
rs9930333	Codominant	T/T	362 (72.7%)	352 (70.7%)	1	0.210	1371.9	1406.2
		G/T	119 (23.9%)	136 (27.3%)	0.85 (0.64–1.14)			
		G/G	17 (3.4%)	10 (2%)	1.66 (0.75–3.70)			
	Dominant	T/T	362 (72.7%)	352 (70.7%)	1	0.490	1372.5	1401.9
		G/T-G/G	136 (27.3%)	146 (29.3%)	0.91 (0.68–1.20)			
	Recessive	T/T-G/T	481 (96.6%)	488 (98%)	1	0.170	1371.1	1400.5
		G/G	17 (3.4%)	10 (2%)	1.74 (0.78–3.85)			
	Log-additive	–	–	–	0.98 (0.77–1.25)	0.860	1373	1402.4
rs11642015	Codominant	C/C	397 (79.7%)	397 (79.7%)	1	0.890	1374.8	1409.1
		T/C	93 (18.7%)	95 (19.1%)	0.99 (0.72–1.37)			
		T/T	8 (1.6%)	6 (1.2%)	1.30 (0.44–3.83)			
	Dominant	C/C	397 (79.7%)	397 (79.7%)	1	0.940	1373	1402.4
		T/C-T/T	101 (20.3%)	101 (20.3%)	1.01 (0.74–1.38)			
	Recessive	C/C-T/C	490 (98.4%)	492 (98.8%)	1	0.620	1372.8	1402.2
		T/T	8 (1.6%)	6 (1.2%)	1.31 (0.45–3.83)			
	Log-additive	–	–	–	1.03 (0.78–1.36)	0.840	1373	1402.4
rs1558902	Codominant	T/T	400 (80.3%)	398 (79.9%)	1	0.970	1374.9	1409.3
		A/T	91 (18.3%)	94 (18.9%)	0.98 (0.71–1.35)			
		A/A	7 (1.4%)	6 (1.2%)	1.13 (0.37–3.43)			
	Dominant	T/T	400 (80.3%)	398 (79.9%)	1	0.940	1373	1402.4
		A/T-A/A	98 (19.7%)	100 (20.1%)	0.99 (0.72–1.35)			
	Recessive	T/T-A/T	491 (98.6%)	492 (98.8%)	1	0.820	1373	1402.4
		A/A	7 (1.4%)	6 (1.2%)	1.14 (0.38–3.44)			
	Log-additive	–	–	–	1.00 (0.75–1.33)	0.990	1373	1402.4
rs56094641	Codominant	A/A	396 (79.5%)	396 (79.5%)	1	0.890	1374.8	1409.1
		G/A	94 (18.9%)	96 (19.3%)	0.99 (0.72–1.37)			
		G/G	8 (1.6%)	6 (1.2%)	1.30 (0.44–3.83)			
	Dominant	A/A	396 (79.5%)	396 (79.5%)	1	0.950	1373	1402.4
		G/A-G/G	102 (20.5%)	102 (20.5%)	1.01 (0.74–1.38)			
	Recessive	A/A-G/A	490 (98.4%)	492 (98.8%)	1	0.620	1372.8	1402.2
		G/G	8 (1.6%)	6 (1.2%)	1.31 (0.45–3.83)			
	Log-additive	–	–	–	1.03 (0.77–1.36)	0.850	1373	1402.4
rs3751812	Codominant	G/G	397 (80%)	396 (79.5%)	1	0.960	1372.7	1407
		G/T	92 (18.6%)	96 (19.3%)	0.98 (0.71–1.35)			
		T/T	7 (1.4%)	6 (1.2%)	1.14 (0.38–3.44)			
	Dominant	G/G	397 (80%)	396 (79.5%)	1	0.920	1370.7	1400.1
		G/T-T/T	99 (20%)	102 (20.5%)	0.98 (0.72–1.35)			
	Recessive	G/G-G/T	489 (98.6%)	492 (98.8%)	1	0.810	1370.7	1400.1
		T/T	7 (1.4%)	6 (1.2%)	1.14 (0.38–3.45)			
	Log-additive	–	–	–	1.00 (0.75–1.32)	0.980	1370.7	1400.2
rs62033406	Codominant	A/A	126 (25.4%)	164 (32.9%)	1	**0.011**	1364.7	1399
		G/A	262 (52.7%)	236 (47.4%)	**1.54 (1.14–2.07)**			
		G/G	109 (21.9%)	98 (19.7%)	**1.52 (1.06–2.18)**			
	Dominant	A/A	126 (25.4%)	164 (32.9%)	1	0.003*	1362.7	1392.1
		G/A-G/G	371 (74.7%)	334 (67.1%)	**1.53 (1.16–2.03)**			
	Recessive	A/A-G/A	388 (78.1%)	400 (80.3%)	1	0.350	1370.8	1400.2
		G/G	109 (21.9%)	98 (19.7%)	1.16 (0.85–1.58)			
	Log-additive	–	–	–	**1.25 (1.05–1.50)**	**0.014**	1365.5	1394.9

ONFH, osteonecrosis of the femoral head; SNP, single nucleotide polymorphism; OR, odds ratio; 95% CI, 95% confidence interval; AIC, akaike information criterion; BIC, Bayesian information criterion

*p* values were calculated using logistic regression analysis adjusted by gender, age, smoking and drinking

Bold indicate that *p* < 0.05 means the data is statistically significant

^*^p indicate that after Bonferroni correction (*p* < 0.05/6) means the data is statistically significant

### Stratification analysis for the contribution of FTO SNPs to ONFH risk

**Fig. 1:**
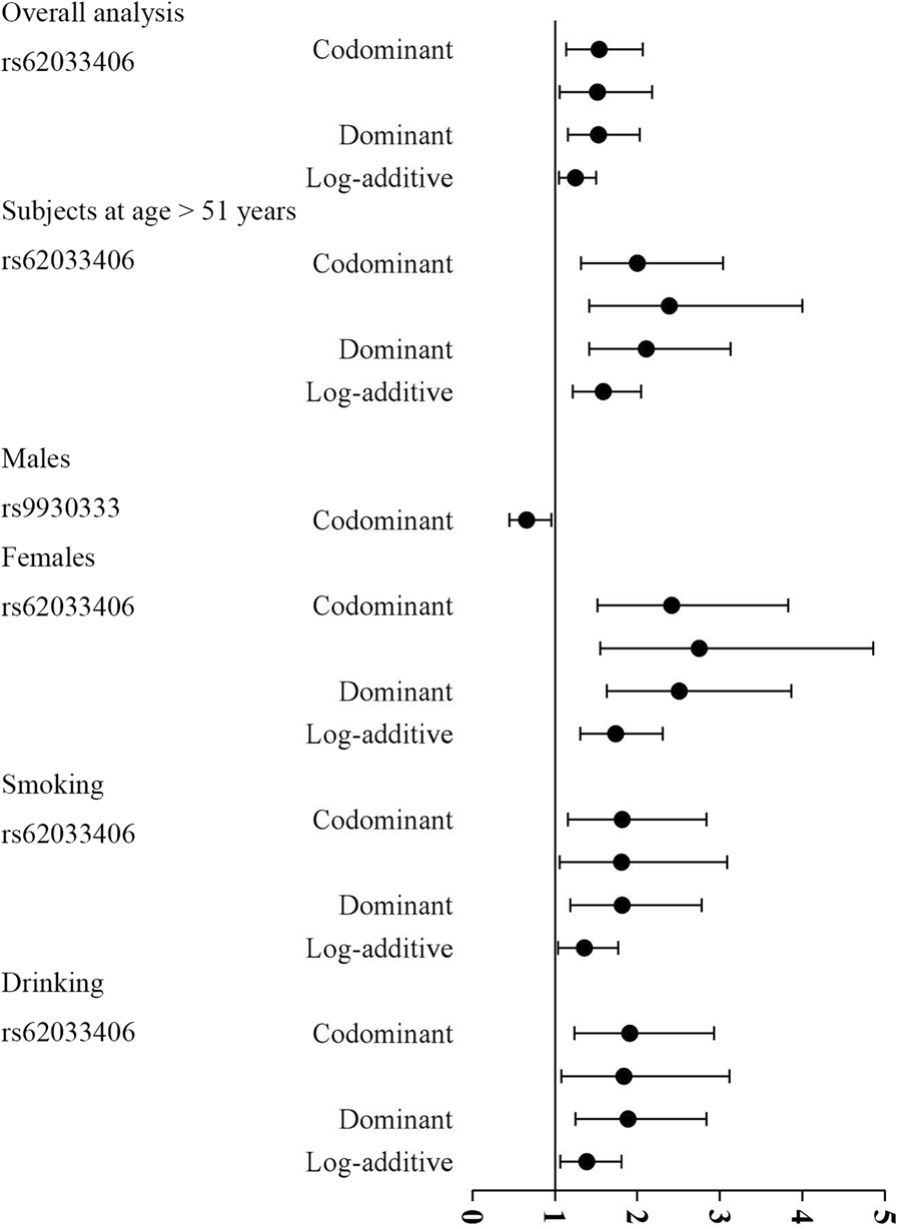
Forest plot of *FTO* polymorphisms and ONFH susceptibility. The horizontal lines represent the study-specific ORs and 95% CIs

To identify subgroup specific associations, we performed stratification analysis based on the major risk factors, such as age, sex, smoking, alcohol drinking and clinical stages (Table 4; Additional file 1: Tables S2, and S3). The results displayed that *FTO* rs62033406-G was related to the risk of ONFH in the subgroup at age > 51 years (codominant: OR = 2.00, OR = 2.39, *p* = 7.00 × 10^–4^; dominant: OR = 2.11, *p* = 2.00 × 10^–4^; and log-additive: OR = 1.25, *p* = 4.00 × 10^–4^), females (codominant: OR = 2.42, OR = 2.75, *p* = 1.00 × 10^–4^; dominant: OR = 2.51, *p* < 0.0001; and log-additive: OR = 1.74, *p* = 1.00 × 10^–4^), smokers (codominant: OR = 1.82, OR = 1.81, *p* = 0.020; dominant: OR = 1.82, *p* = 0.005; and log-additive: OR = 1.36, *p* = 0.023) and drinkers (codominant: OR = 1.91, OR = 1.84,* p* = 0.009; dominant: OR = 1.89, *p* = 0.002; and log-additive: OR = 1.39, *p* = 0.014), respectively. After Bonferroni correction, rs62033406-G was associated with an increased incidence in the subgroup at age > 51 years (codominant, dominant, and log-additive), females (codominant, dominant, and log-additive), smokers (dominant), and drinkers (dominant), respectively. Besides, rs9930333 GT genotype was related to a reduced risk of ONFH compared to the TT genotype (OR = 0.66, *p* = 0.030). However, no significant relationship of selected polymorphisms to ONFH in the stratified analysis by clinical stages was found (Additional file 1: Table S3). These results of overall analysis and stratification analysis was shown in Fig. 1.

**Table 4:** Association between *FTO* polymorphisms and ONFH risk according to the stratification analysis

SNP ID	Model	Genotype	Case	Control	OR (95% CI)	*P* value	Control	Case	OR (95% CI)	*P* value
Age, years		> 51					≤ 51			
rs62033406	Codominant	A/A	64 (23.4%)	88 (37.3%)	1	7.00 × 10^–4*^	62 (27.7%)	76 (29%)	1	0.650
		G/A	143 (52.4%)	108 (45.8%)	**2.00 (1.32–3.04)**		119 (53.1%)	128 (48.9%)	1.15 (0.76–1.76)	
		G/G	66 (24.2%)	40 (16.9%)	**2.39 (1.42–4.00)**		43 (19.2%)	58 (22.1%)	0.95 (0.56–1.59)	
	Dominant	A/A	64 (23.4%)	88 (37.3%)	1	2.00 × 10^–4*^	62 (27.7%)	76 (29%)	1	0.680
		G/A-G/G	209 (76.6%)	148 (62.7%)	**2.11 (1.42–3.13)**		162 (72.3%)	186 (71%)	1.09 (0.73–1.62)	
	Recessive	A/A-G/A	207 (75.8%)	196 (83%)	1	0.051	181 (80.8%)	204 (77.9%)	1	0.510
		G/G	66 (24.2%)	40 (16.9%)	1.55 (0.99–2.42)		43 (19.2%)	58 (22.1%)	0.86 (0.55–1.35)	
	Log-additive	–	–	–	**1.59 (1.22–2.05)**	4.00 × 10^–4*^	–	–	0.99 (0.76–1.28)	0.910
Gender		Males					Females			
rs9930333	Codominant	T/T	206 (73.6%)	209 (67.2%)	1	0.030	156 (71.6%)	143 (76.5%)	1	0.650
		G/T	63 (22.5%)	96 (30.9%)	**0.66 (0.45–0.96)**		56 (25.7%)	40 (21.4%)	1.24 (0.77–1.99)	
		G/G	11 (3.9%)	6 (1.9%)	1.86 (0.67–5.16)		6 (2.8%)	4 (2.1%)	1.24 (0.34–4.56)	
	Dominant	T/T	206 (73.6%)	209 (67.2%)	1	0.081	156 (71.6%)	143 (76.5%)	1	0.350
		G/T-G/G	74 (26.4%)	102 (32.8%)	0.73 (0.51–1.04)		62 (28.4%)	44 (23.5%)	1.24 (0.79–1.96)	
	Recessive	T/T-G/T	269 (96.1%)	305 (98.1%)	1	0.150	212 (97.2%)	183 (97.9%)	1	0.810
		G/G	11 (3.9%)	6 (1.9%)	2.08 (0.75–5.76)		6 (2.8%)	4 (2.1%)	1.17 (0.32–4.29)	
	Log-additive	–	–	–	0.85 (0.62–1.16)	0.290	–	–	1.20 (0.80–1.79)	0.370
rs62033406	Codominant	A/A	74 (26.5%)	82 (26.4%)	1	0.820	52 (23.9%)	82 (43.9%)	1	1.00 × 10^–4*^
		G/A	150 (53.8%)	162 (52.1%)	1.09 (0.73–1.60)		112 (51.4%)	74 (39.6%)	**2.42 (1.52–3.83)**	
		G/G	55 (19.7%)	67 (21.5%)	0.96 (0.59–1.55)		54 (24.8%)	31 (16.6%)	**2.75 (1.55–4.86)**	
	Dominant	A/A	74 (26.5%)	82 (26.4%)	1	0.810	52 (23.9%)	82 (43.9%)	1	< 0.0001*
		G/A-G/G	205 (73.5%)	229 (73.6%)	1.05 (0.72–1.52)		166 (76.2%)	105 (56.1%)	**2.51 (1.63–3.87)**	
	Recessive	A/A-G/A	224 (80.3%)	244 (78.5%)	1	0.630	164 (75.2%)	156 (83.4%)	1	0.049
		G/G	55 (19.7%)	67 (21.5%)	0.90 (0.60–1.36)		54 (24.8%)	31 (16.6%)	1.65 (1.00–2.72)	
	Log-additive	–	–	–	0.98 (0.77–1.25)	0.890	–	–	**1.74 (1.31–2.31)**	1.00 × 10^–4*^
Smoking			Smokers				Non-smokers			
rs62033406	Codominant	A/A	44 (20.1%)	82 (30.5%)	1	0.020	82 (29.5%)	82 (35.8%)	1	0.290
		G/A	125 (57.1%)	134 (49.8%)	**1.82 (1.16–2.84)**		137 (49.3%)	102 (44.5%)	1.36 (0.91–2.03)	
		G/G	50 (22.8%)	53 (19.7%)	**1.81 (1.06–3.09)**		59 (21.2%)	45 (19.6%)	1.34 (0.81–2.22)	
	Dominant	A/A	44 (20.1%)	82 (30.5%)	1	0.005*	82 (29.5%)	82 (35.8%)	1	0.120
		G/A-G/G	175 (79.9%)	187 (69.5%)	**1.82 (1.19–2.78)**		196 (70.5%)	147 (64.2%)	1.35 (0.93–1.98)	
	Recessive	A/A-G/A	169 (77.2%)	216 (80.3%)	1	0.410	219 (78.8%)	184 (80.3%)	1	0.610
		G/G	50 (22.8%)	53 (19.7%)	1.20 (0.78–1.87)		59 (21.2%)	45 (19.6%)	1.12 (0.72–1.74)	
	Log-additive	–	–	–	**1.36 (1.04–1.77)**	0.023	–	–	1.18 (0.92–1.51)	0.190
Drinking			Drinkers				Non-drinkers			
rs62033406	Codominant	A/A	58 (21.9%)	81 (31.6%)	1	0.009	68 (29.3%)	83 (34.3%)	1	0.460
		G/A	150 (56.6%)	127 (49.6%)	**1.91 (1.24–2.93)**		112 (48.3%)	109 (45%)	1.27 (0.84–1.94)	
		G/G	57 (21.5%)	48 (18.8%)	**1.84 (1.08–3.12)**		52 (22.4%)	50 (20.7%)	1.29 (0.78–2.15)	
	Dominant	A/A	58 (21.9%)	81 (31.6%)	1	0.002*	68 (29.3%)	83 (34.3%)	1	0.210
		G/A-G/G	207 (78.1%)	175 (68.4%)	**1.89 (1.25–2.84)**		164 (70.7%)	159 (65.7%)	1.28 (0.87–1.89)	
	Recessive	A/A-G/A	208 (78.5%)	208 (81.2%)	1	0.430	180 (77.6%)	192 (79.3%)	1	0.610
		G/G	57 (21.5%)	48 (18.8%)	1.19 (0.77–1.86)		52 (22.4%)	50 (20.7%)	1.12 (0.72–1.74)	
	Log-additive	–	–	–	**1.39 (1.07–1.81)**	0.014	–	–	1.15 (0.89–1.48)	0.280

ONFH, osteonecrosis of the femoral head; SNP, single nucleotide polymorphism; OR, odds ratio; 95% CI, 95% confidence interval

*p* values were calculated using logistic regression analysis adjusted by gender, age, smoking and/or drinking

Bold indicate that *p* < 0.05 means the data is statistically significant

^*^p indicate that after Bonferroni correction (*p* < 0.05/6) means the data is statistically significant

### FPRP analysis for the positive findings

FPRP analysis was performed to interrogate whether the significant findings were deserving attention (Table 5). At the prior probability level of 0.1, the significant association for rs62033406-G (FPRP = 0.081, 0.061, and 0.132) remained noteworthy in the overall analysis. Moreover, the associations of rs62033406-G with ONFH susceptibility were also positive in the subgroup at age > 51 years at the prior probability level of 0.01, females at the prior probability level of 0.001, and smokers and drinkers at the prior probability level of 0.1, respectively.

**Table 5:** False-positive report probability for the associations of *FTO* polymorphisms with ONFH risk

Group/ SNPs ID	Model	OR (95% CI)	Prior probability				
			0.25	0.1	0.01	0.001	0.0001
Overall							
rs62033406	Codominant	1.54 (1.14–2.07)	**0.029**	**0.081**	0.492	0.907	0.990
		1.52 (1.06–2.18)	**0.127**	0.304	0.828	0.980	0.998
	Dominant	1.53 (1.16–2.03)	**0.021**	**0.061**	0.416	0.878	0.986
	Log-additive	1.25 (1.05–1.50)	**0.048**	**0.132**	0.625	0.944	0.994
Age > 51 years							
rs62033406	Codominant	2.00 (1.32–3.04)	**0.007**	**0.021**	**0.189**	0.701	0.959
		2.39 (1.42–4.00)	**0.011**	**0.032**	0.266	0.786	0.973
	Dominant	2.11 (1.42–3.13)	**0.002**	**0.005**	**0.049**	0.343	0.839
	Log-additive	1.59 (1.22–2.05)	**0.003**	**0.009**	**0.095**	0.515	0.914
Males							
rs9930333	Codominant	0.66 (0.45–0.96)	**0.157**	0.358	0.860	0.984	0.998
Females							
rs62033406	Codominant	2.42 (1.52–3.83)	**0.002**	**0.007**	**0.071**	0.437	0.886
		2.75 (1.55–4.86)	**0.011**	**0.032**	0.265	0.785	0.973
	Dominant	2.51 (1.63–3.87)	**0.001**	**0.002**	**0.020**	**0.169**	0.671
	Log-additive	1.74 (1.31–2.31)	**0.003**	**0.007**	**0.077**	0.455	0.893
Smokers			**0.036**	**0.102**	0.556	0.927	0.992
rs62033406	Codominant	1.82 (1.16–2.84)	**0.122**	0.294	0.821	0.979	0.998
		1.81 (1.06–3.09)	**0.024**	**0.070**	0.453	0.893	0.988
	Dominant	1.82 (1.19–2.78)	**0.080**	0.207	0.741	0.967	0.997
	Log-additive	1.36 (1.04–1.77)					
Drinkers							
rs62033406	Codominant	1.91 (1.24–2.93)	**0.015**	**0.045**	0.340	0.839	0.981
		1.84 (1.08–3.12)	**0.102**	0.255	0.790	0.974	0.997
	Dominant	1.89 (1.25–2.84)	**0.011**	**0.031**	0.263	0.782	0.973
	Log-additive	1.39 (1.07–1.81)	**0.042**	**0.116**	0.590	0.936	0.993

SNP, single nucleotide polymorphism; OR, odds ratio; 95% CI, 95% confidence interval

The level of false-positive report probability threshold was set at 0.2, and noteworthy findings are presented

Bold indicate that the level of false-positive report probability threshold < 0.2

### MDR analysis for the contribution of FTO SNPs to ONFH risk

**Fig. 2:**
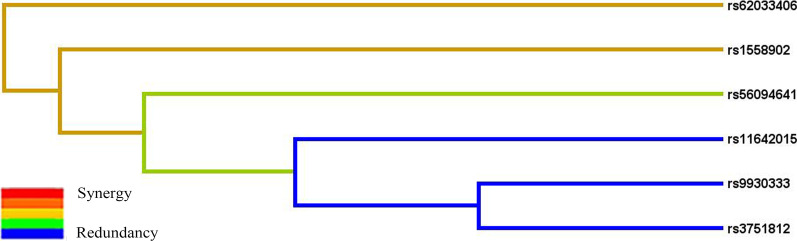
Dendrogram for the interactions among *FTO* SNPs on the risk of ONFH. Short connections among nodes represent stronger interactions

**Fig. 3:**
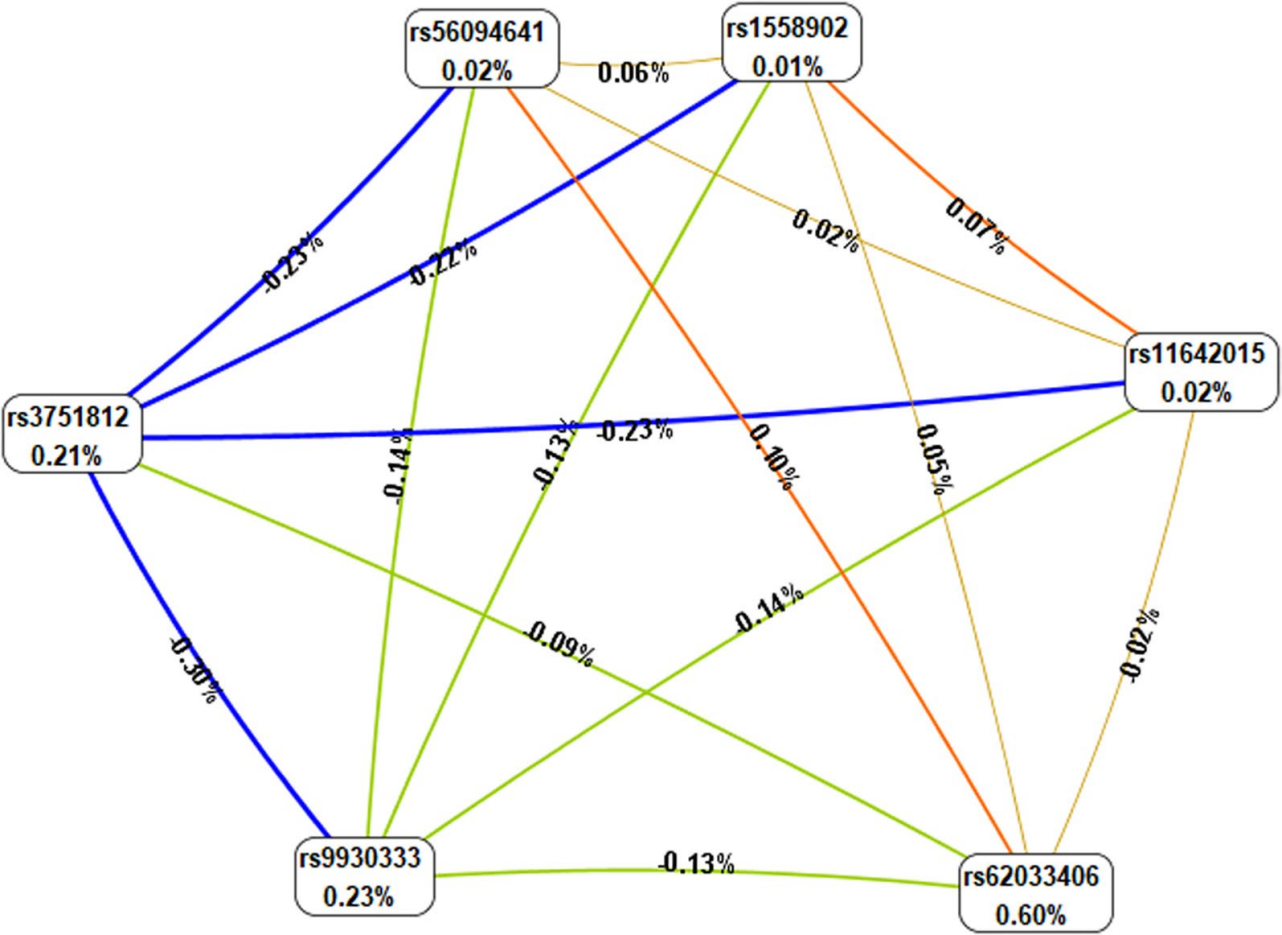
Fruchterman-Reingold plot of *FTO* SNP-SNP interaction for ONFH susceptibility. Values in nodes represent the IGs of individual attribute (main effects). Values between nodes are IGs of each pair of attributes (interaction effects)

MDR analysis was applied to evaluate the effect of SNP-SNP interaction on ONFH risk (Figs. 2, 3; Table 6). Table 6 displayed the results for one- to six-locus models. Rs62033406 A>G was the best single–locus model for ONFH risk, with the highest testing accuracy (0.5382, *p* = 0.008) and perfect cross-validation consistency (10/10). The best multi–loci model was the five–loci model, a combination of rs9930333 T>G, rs1558902 T>A, rs56094641 A>G, rs3751812 G>T, and rs62033406 A>G (testing accuracy, 0.5351; *p* = 0.0004; cross–validation consistency, 10/10). The dendrogram plot (Fig. 2) described the SNP-SNP interactions and recapitulated the main and/or interaction effect for each pairwise combination of attributes. The fruchterman-Reingold plot (Fig. 3) revealed that rs62033406 had the information gain (0.60%) of individual attribute regarding ONFH.

**Table 6:** MDR analysis for SNP–SNP interaction in *FTO* with ONFH susceptibility

Model	Training Bal. Acc	Testing Bal. Acc	CVC	OR (95% CI)	*p*
rs62033406	0.5382	0.5382	10/10	1.45 (1.10–1.91)	**0.008**
rs56094641, rs62033406	0.5486	0.5321	8/10	1.52 (1.17–1.97)	**0.0016**
rs56094641, rs3751812, rs62033406	0.5522	0.5341	5/10	1.56 (1.20–2.02)	**0.0008**
rs9930333, rs56094641, rs3751812, rs62033406	0.5541	0.5351	7/10	1.59 (1.22–2.06)	**0.0005**
rs9930333, rs1558902, rs56094641, rs3751812, rs62033406	0.5558	0.5351	10/10	1.60 (1.24–2.08)	**0.0004**
rs9930333, rs11642015, rs1558902, rs56094641, rs3751812, rs62033406	0.5558	0.5351	10/10	1.60 (1.24–2.08)	**0.0004**

MDR, multifactor dimensionality reduction; SNP, single nucleotide polymorphism; ONFH, osteonecrosis of the femoral head; Bal. Acc., balanced accuracy; CVC, cross–validation consistency; OR, odds ratio; CI, confidence interval

*p* values were calculated using χ^2^ tests

Bold indicate that *p* < 0.05 indicates statistical significance

## Discussion

Here, six SNPs in *FTO* were examined in 996 participants to investigate the susceptibility to ONFH risk in the Chinese Han population. This study was the first to demonstrate that rs62033406 A>G contributed to an increased risk of ONFH under the allele, genotype, dominant and log-additive models. Specially, *FTO* rs62033406 A > G was related to ONFH susceptibility in the subgroup at age > 51 years, females, smokers, and drinkers, respectively. MDR analysis displayed that rs62033406 A>G was the best single–locus model for ONFH risk, and the best multi–loci model was the five-locus model, a combination of rs9930333 T>G, rs1558902 T>A, rs56094641 A>G, rs3751812 G>T, and rs62033406 A>G. These results indicate that *FTO* SNPs may have an important role in ONFH risk among the Han Chinese population, which have provided insights into ONFH occurrence.

*FTO* mRNA was downregulated in osteonecrosis patients. During osteogenic differentiation, *FTO* induced the expression of osteoblast biomarkers ALPL and OPN [13]. Some recent studies suggested that genetic polymorphisms of *FTO* might affect the expression of the adjacent genes and were associated with disease [19]. However, no studies have evaluated the impact of *FTO* polymorphisms on ONFH risk. Here, six SNPs (rs9930333 T>G, rs11642015 C>T, rs1558902 T>A, rs56094641 A>G, rs3751812 G>T, and rs62033406 A>G) in the *FTO* gene were genotyped to assess the risk-association with ONFH occurrence. HaploReg v4.1 database displayed that these SNPs might may be associated with promoter/Enhancer histone marks, DNAse, proteins bound, and motifs changed [20]. This study was the first to demonstrate that rs62033406 A>G contributed to an increased risk of ONFH in the Chinese Han population after Bonferroni correction. Several studies provided increasing evidence to support that intronic SNPs confer susceptibilities by affecting gene expression [21, 22]. SNPs within intron of the *FTO* gene are related to *FTO* transcript levels [23]. In *silico*, rs62033406 A>G located in the intronic regions of the *FTO* gene could be as a possible enhancer activity or motifs change, which may affect the activity or expression of *FTO* and may change the motif of functional DNA binding sites and thus impact the regulation of transcription and alternative splicing in ONFH occurrence. However, the hypothesis required requires further experimental confirmation. In future, we would design detailed experiments to further explore the association between rs62033406 A>G and *FTO* expression and the biological functions of rs62033406 in ONFH occurrence.

ONFH primarily affects young to middle‐aged adults, the mean age at diagnosis is 48 years in China, with majority of their patients were between 30 and 65 years [24]. Given that age is the risk factor for ONFH, we further explored the effect of *FTO* variant on susceptibility to ONFH in the stratification analysis by age. A study reported that the median of ONFH patients was 50 years [25]. Besides, the mean ages of ONFH patients and the controls in the study were 51.77 ± 14.55 years and 50.38 ± 14.52 years, respectively. To explore the contribution of age, we divided the cases and controls into two groups as ≤ 51 years and > 51 years, respectively. The results displayed that *FTO* rs62033406 A>G was related to the risk of ONFH in the subgroup at age > 51 years but not in the subjects with age ≤ 51 years, which indicated that the influence of rs62033406 A>G on ONFH susceptibility presented age difference. Moreover, the prevalence rate was higher in men than in women in China [2]. We noticed that rs62033406 A>G affected ONFH risk in females but not in males, suggesting that genetic susceptibility to ONFH might differ by sex. Specially, rs62033406 A>G was associated with the higher ONFH risk after Bonferroni correction in the subgroup at age > 51 years and in females. The differential genetic association pattern in subjects aged > 51 years and in females might be influenced by their hormonal, genetic, and behavioral factors.

Smoking and alcohol intake are modifiable lifestyle-related risk factors for ONFH [26]. A meta-analysis showed that smokers were at a higher risk of ONFH [27]. Alcohol intake is positively related to an increased risk of ONFH in a non-linear pattern [28]. Specially, *FTO* rs62033406 A>G was related to the risk of ONFH in smokers and drinkers, but not in non-smokers and non-drinkers respectively. After Bonferroni correction, rs62033406 A>G was associated with an increased incidence in smokers and drinkers under the dominant model. These findings emphasize the importance of considering gene-behavioral habit in association studies between genetic and ONFH risk. These results might emphasize the importance of considering heterogeneity in genetic and ONFH association studies.

Given that ONFH is a complex polygenic disease, SNP-SNP interaction studies may help to discover the risk factors of ONFH. Of note, MDR is a powerful method to detect SNP-SNP interactions without main gene effects for complex diseases in case–control studies [29]. Further, the MDR was used to analyses the interactions of these six SNPs, the result suggested that rs62033406 A>G was the best single–locus model for ONFH risk, which was consistent with the results of logistic regression analysis. The fruchterman-Reingold revealed that rs62033406 had the information gain (0.60%) of individual attribute regarding ONFH. The best multi–loci model was the five-locus model, a combination of rs9930333 T>G, rs1558902 T>A, rs56094641 A>G, rs3751812 G>T, and rs62033406 A>G, which might further support the multi-factor model contributing to ONFH occurrence. Furthermore, rs62033406 was found to have a significant combinatorial effect with four other SNPs. This hints that the combination of high-risk genotypes carrying these variants may have important effect on ONFH pathogenesis. The complexity of the genetic interaction network in ONFH occurrence need to further investigate.

However, some intrinsic limitations should be considered. First, all participants are limited to the Han Chinese population, therefore, whether our finding are applicable to other populations requires further research to confirm. Second, this is a hospital-based, single-center study, so the selection bias cannot be excluded. Third, the mechanism of *FTO* rs62033406 A>G on the occurrence of ONFH is still unclear, and the analysis of biological functions is needed to further research. Despite the above limitations, our research results provide scientific evidence for the impact of *FTO* on the risk of ONFH in future studies.

## Conclusion

To sum up, our results provide new light on the contribution of *FTO* polymorphisms to ONFH susceptibility among the Chinese Han population, which may offer the new candidate genes for elucidating the pathogenesis of ONFH. However, larger-scale prospective studies and functional studies are needed to confirm our results and clarify the underlying mechanism that *FTO* polymorphisms confers susceptibility to ONFH.

## Supplementary Information

**Additional file 1.** **Suppl_Table 1.** Primers sequence of PCR and UEP for FTO SNPs. **Suppl_Table 2.** Association between FTO polymorphisms and ONFH risk according to the stratification analysis. **Suppl_Table 3.** Association between FTO polymorphisms and ONFH risk in the stratified analysis by clinical stages.

## Data Availability

Data supporting the findings of this study are available from the Corresponding Author (Bing Zhu), but their availability is limited, they are used under the license of the current study and are therefore not publicly available. However, upon reasonable request, authors may provide data.
