# Supplementary material for: FTO rs62033406 A>G associated with the risk of osteonecrosis of the femoral head among the Chinese Han population
Source: BMC Med Genomics. 2022 Jun 15;15:132. doi: 10.1186/s12920-022-01283-z (PMC9202150; doi:10.1186/s12920-022-01283-z)
Supplement: Supplementary file 1 — Additional file 1. Suppl_Table 1. Primers sequence of PCR and UEP for FTO SNPs. Suppl_Table 2. Association between FTO polymorphisms and ONFH risk according to the stratification analysis. Suppl_Table 3. Association between FTO polymorphisms and ONFH risk in the stratified analysis by clinical stages. [file 12920_2022_1283_MOESM1_ESM.docx]

**Suppl_Table 1. Primers sequence of PCR and UEP for *FTO*** **SNPs**

| **SNPs** | **First Primer(5'-3')** | **Second Primer (5'-3')** | **UEP_DIR** | | **UEP SEQ (5'-3')** |
| --- | --- | --- | --- | --- | --- |
| rs9930333 | ACGTTGGATGAGGCCAGAGTAGAATTAGGG | ACGTTGGATGTTCCCCTTCAGTCTATCTGC | | F | tcccGTTCTGATGGCTTGGCCCAG |
| rs11642015 | ACGTTGGATGTATCCATCCCACCAAGGTCC | ACGTTGGATGACCCGAGTAGAAGATGATGC | | R | aCCACCAAGGTCCTGCTACAC |
| rs1558902 | ACGTTGGATGCTACAGGCATTGTGTCTAGC | ACGTTGGATGCAAGTTAGGGTACGTTGCAG | | F | gGCCCTGTGGGTTTACATTAG |
| rs56094641 | ACGTTGGATGCAAAGCCTAGTTACTGCCTC | ACGTTGGATGCTCTGTATCATCAACATGTG | | R | GCCTCATGGAGTTTACATTTTAA |
| rs3751812 | ACGTTGGATGCTTTTTCGCTGGTAGGATGC | ACGTTGGATGAGACCTGAAAATAGGTGAGC | | R | ggGCCTCTCCCTGCCAACA |
| rs62033406 | ACGTTGGATGTAATGTCGTCACTAGCCAGC | ACGTTGGATGTTGTTTCTAGCTTCCCCCAG | | R | gGAATTTGTAGAGACAGAAAATAGAA |

SNP: Single nucleotide polymorphism; UEP: Unextended mini sequencing primer; SEQ, sequence.

**Suppl_Table 2.** Association between ***FTO*** polymorphisms and **ONFH risk** according to the stratification analysis

| SNP ID | Model | Genotype | Case | Control | OR (95% CI) | *p*-value | Control | Case | OR (95% CI) | *p*-value |
| --- | --- | --- | --- | --- | --- | --- | --- | --- | --- | --- |
| **Age, years** |  | **> 51** | | | | | **≤ 51** | | | |
| rs9930333 | Codominant | T/T | 173 (73.3%) | 198 (72.5%) | 1 | 0.540 | 179 (68.3%) | 164 (72.9%) | 1 | 0.270 |
|  |  | G/T | 58 (24.6%) | 65 (23.8%) | 0.95 (0.63-1.43) |  | 78 (29.8%) | 54 (24%) | 0.75 (0.50-1.13) |  |
|  |  | G/G | 5 (2.1%) | 10 (3.7%) | 1.78 (0.59-5.38) |  | 5 (1.9%) | 7 (3.1%) | 1.49 (0.46-4.83) |  |
|  | Dominant | T/T | 173 (73.3%) | 198 (72.5%) | 1 | 0.950 | 179 (68.3%) | 164 (72.9%) | 1 | 0.250 |
|  |  | G/T-G/G | 63 (26.7%) | 75 (27.5%) | 1.01 (0.68-1.51) |  | 83 (31.7%) | 61 (27.1%) | 0.79 (0.53-1.18) |  |
|  | Recessive | T/T-G/T | 231 (97.9%) | 263 (96.3%) | 1 | 0.280 | 257 (98.1%) | 218 (96.9%) | 1 | 0.420 |
|  |  | G/G | 5 (2.1%) | 10 (3.7%) | 1.80 (0.60-5.43) |  | 5 (1.9%) | 7 (3.1%) | 1.62 (0.50-5.20) |  |
|  | Log-additive | --- | --- | --- | 1.07 (0.76-1.51) | 0.690 | --- | --- | 0.87 (0.61-1.24) | 0.440 |
| rs11642015 | Codominant | C/C | 197 (83.5%) | 215 (78.8%) | 1 | 0.440 | 200 (76.3%) | 182 (80.9%) | 1 | 0.340 |
|  |  | T/C | 35 (14.8%) | 53 (19.4%) | 1.36 (0.85-2.19) |  | 60 (22.9%) | 40 (17.8%) | 0.74 (0.47-1.16) |  |
|  |  | T/T | 4 (1.7%) | 5 (1.8%) | 1.12 (0.29-4.31) |  | 2 (0.8%) | 3 (1.3%) | 1.65 (0.27-10.00) |  |
|  | Dominant | C/C | 197 (83.5%) | 215 (78.8%) | 1 | 0.210 | 200 (76.3%) | 182 (80.9%) | 1 | 0.240 |
|  |  | T/C-T/T | 39 (16.5%) | 58 (21.2%) | 1.34 (0.85-2.11) |  | 62 (23.7%) | 43 (19.1%) | 0.77 (0.49-1.19) |  |
|  | Recessive | C/C-T/C | 232 (98.3%) | 268 (98.2%) | 1 | 0.930 | 260 (99.2%) | 222 (98.7%) | 1 | 0.540 |
|  |  | T/T | 4 (1.7%) | 5 (1.8%) | 1.06 (0.27-4.08) |  | 2 (0.8%) | 3 (1.3%) | 1.75 (0.29-10.61) |  |
|  | Log-additive | --- | --- | --- | 1.26 (0.84-1.88) | 0.260 | --- | --- | 0.82 (0.54-1.23) | 0.340 |
| rs1558902 | Codominant | T/T | 197 (83.5%) | 216 (79.1%) | 1 | 0.410 | 201 (76.7%) | 184 (81.8%) | 1 | 0.270 |
|  |  | A/T | 35 (14.8%) | 53 (19.4%) | 1.37 (0.85-2.20) |  | 59 (22.5%) | 38 (16.9%) | 0.71 (0.45-1.12) |  |
|  |  | A/A | 4 (1.7%) | 4 (1.5%) | 0.89 (0.21-3.69) |  | 2 (0.8%) | 3 (1.3%) | 1.64 (0.27-9.94) |  |
|  | Dominant | T/T | 197 (83.5%) | 216 (79.1%) | 1 | 0.230 | 201 (76.7%) | 184 (81.8%) | 1 | 0.180 |
|  |  | A/T-A/A | 39 (16.5%) | 57 (20.9%) | 1.32 (0.84-2.08) |  | 61 (23.3%) | 41 (18.2%) | 0.74 (0.47-1.15) |  |
|  | Recessive | T/T-A/T | 232 (98.3%) | 269 (98.5%) | 1 | 0.810 | 260 (99.2%) | 222 (98.7%) | 1 | 0.540 |
|  |  | A/A | 4 (1.7%) | 4 (1.5%) | 0.84 (0.20-3.49) |  | 2 (0.8%) | 3 (1.3%) | 1.75 (0.29-10.61) |  |
|  | Log-additive | --- | --- | --- | 1.23 (0.82-1.84) | 0.320 | --- | --- | 0.79 (0.52-1.20) | 0.270 |
| rs56094641 | Codominant | A/A | 197 (83.5%) | 214 (78.4%) | 1 | 0.390 | 199 (76%) | 182 (80.9%) | 1 | 0.290 |
|  |  | G/A | 35 (14.8%) | 54 (19.8%) | 1.39 (0.87-2.23) |  | 61 (23.3%) | 40 (17.8%) | 0.72 (0.46-1.13) |  |
|  |  | G/G | 4 (1.7%) | 5 (1.8%) | 1.12 (0.29-4.33) |  | 2 (0.8%) | 3 (1.3%) | 1.64 (0.27-9.95) |  |
|  | Dominant | A/A | 197 (83.5%) | 214 (78.4%) | 1 | 0.180 | 199 (76%) | 182 (80.9%) | 1 | 0.200 |
|  |  | G/A-G/G | 39 (16.5%) | 59 (21.6%) | 1.36 (0.86-2.15) |  | 63 (24.1%) | 43 (19.1%) | 0.75 (0.48-1.16) |  |
|  | Recessive | A/A-G/A | 232 (98.3%) | 268 (98.2%) | 1 | 0.930 | 260 (99.2%) | 222 (98.7%) | 1 | 0.540 |
|  |  | G/G | 4 (1.7%) | 5 (1.8%) | 1.06 (0.27-4.08) |  | 2 (0.8%) | 3 (1.3%) | 1.75 (0.29-10.61) |  |
|  | Log-additive | --- | --- | --- | 1.28 (0.86-1.91) | 0.230 | --- | --- | 0.80 (0.53-1.21) | 0.290 |
| rs3751812 | Codominant | G/G | 197 (83.5%) | 214 (79%) | 1 | 0.380 | 199 (76%) | 183 (81.3%) | 1 | 0.240 |
|  |  | G/T | 35 (14.8%) | 53 (19.6%) | 1.39 (0.86-2.23) |  | 61 (23.3%) | 39 (17.3%) | 0.70 (0.44-1.10) |  |
|  |  | T/T | 4 (1.7%) | 4 (1.5%) | 0.90 (0.22-3.73) |  | 2 (0.8%) | 3 (1.3%) | 1.63 (0.27-9.90) |  |
|  | Dominant | G/G | 197 (83.5%) | 214 (79%) | 1 | 0.210 | 199 (76%) | 183 (81.3%) | 1 | 0.160 |
|  |  | G/T-T/T | 39 (16.5%) | 57 (21%) | 1.34 (0.85-2.11) |  | 63 (24.1%) | 42 (18.7%) | 0.73 (0.47-1.13) |  |
|  | Recessive | G/G-G/T | 232 (98.3%) | 267 (98.5%) | 1 | 0.820 | 260 (99.2%) | 222 (98.7%) | 1 | 0.540 |
|  |  | T/T | 4 (1.7%) | 4 (1.5%) | 0.85 (0.21-3.52) |  | 2 (0.8%) | 3 (1.3%) | 1.75 (0.29-10.61) |  |
|  | Log-additive | --- | --- | --- | 1.24 (0.83-1.87) | 0.290 | --- | --- | 0.78 (0.52-1.18) | 0.240 |
| **Gender** |  | **Males** | | | | | **Females** | | | |
| rs11642015 | Codominant | C/C | 240 (77.2%) | 226 (80.7%) | 1 | 0.430 | 157 (84%) | 171 (78.4%) | 1 | 0.370 |
|  |  | T/C | 67 (21.5%) | 48 (17.1%) | 0.78 (0.52-1.19) |  | 28 (15%) | 45 (20.6%) | 1.45 (0.85-2.45) |  |
|  |  | T/T | 4 (1.3%) | 6 (2.1%) | 1.40 (0.38-5.11) |  | 2 (1.1%) | 2 (0.9%) | 0.77 (0.10-5.65) |  |
|  | Dominant | C/C | 240 (77.2%) | 226 (80.7%) | 1 | 0.340 | 157 (84%) | 171 (78.4%) | 1 | 0.200 |
|  |  | T/C-T/T | 71 (22.8%) | 54 (19.3%) | 0.82 (0.55-1.23) |  | 30 (16%) | 47 (21.6%) | 1.40 (0.84-2.34) |  |
|  | Recessive | C/C-T/C | 307 (98.7%) | 274 (97.9%) | 1 | 0.560 | 185 (98.9%) | 216 (99.1%) | 1 | 0.750 |
|  |  | T/T | 4 (1.3%) | 6 (2.1%) | 1.47 (0.40-5.33) |  | 2 (1.1%) | 2 (0.9%) | 0.72 (0.10-5.27) |  |
|  | Log-additive | --- | --- | --- | 0.88 (0.62-1.26) | 0.490 | --- | --- | 1.31 (0.81-2.11) | 0.270 |
| rs1558902 | Codominant | T/T | 240 (77.2%) | 229 (81.8%) | 1 | 0.370 | 158 (84.5%) | 171 (78.4%) | 1 | 0.300 |
|  |  | A/T | 67 (21.5%) | 46 (16.4%) | 0.74 (0.49-1.13) |  | 27 (14.4%) | 45 (20.6%) | 1.50 (0.88-2.56) |  |
|  |  | A/A | 4 (1.3%) | 5 (1.8%) | 1.17 (0.31-4.47) |  | 2 (1.1%) | 2 (0.9%) | 0.77 (0.11-5.69) |  |
|  | Dominant | T/T | 240 (77.2%) | 229 (81.8%) | 1 | 0.210 | 158 (84.5%) | 171 (78.4%) | 1 | 0.160 |
|  |  | A/T-A/A | 71 (22.8%) | 51 (18.2%) | 0.77 (0.51-1.16) |  | 29 (15.5%) | 47 (21.6%) | 1.45 (0.86-2.43) |  |
|  | Recessive | T/T-A/T | 307 (98.7%) | 275 (98.2%) | 1 | 0.760 | 185 (98.9%) | 216 (99.1%) | 1 | 0.750 |
|  |  | A/A | 4 (1.3%) | 5 (1.8%) | 1.23 (0.32-4.71) |  | 2 (1.1%) | 2 (0.9%) | 0.72 (0.10-5.27) |  |
|  | Log-additive | --- | --- | --- | 0.82 (0.57-1.19) | 0.290 | --- | --- | 1.35 (0.83-2.19) | 0.220 |
| rs56094641 | Codominant | A/A | 239 (76.8%) | 226 (80.7%) | 1 | 0.380 | 157 (84%) | 170 (78%) | 1 | 0.300 |
|  |  | G/A | 68 (21.9%) | 48 (17.1%) | 0.77 (0.51-1.16) |  | 28 (15%) | 46 (21.1%) | 1.49 (0.88-2.53) |  |
|  |  | G/G | 4 (1.3%) | 6 (2.1%) | 1.40 (0.38-5.09) |  | 2 (1.1%) | 2 (0.9%) | 0.77 (0.11-5.69) |  |
|  | Dominant | A/A | 239 (76.8%) | 226 (80.7%) | 1 | 0.290 | 157 (84%) | 170 (78%) | 1 | 0.160 |
|  |  | G/A-G/G | 72 (23.1%) | 54 (19.3%) | 0.81 (0.54-1.20) |  | 30 (16%) | 48 (22%) | 1.44 (0.86-2.41) |  |
|  | Recessive | A/A-G/A | 307 (98.7%) | 274 (97.9%) | 1 | 0.560 | 185 (98.9%) | 216 (99.1%) | 1 | 0.750 |
|  |  | G/G | 4 (1.3%) | 6 (2.1%) | 1.47 (0.40-5.33) |  | 2 (1.1%) | 2 (0.9%) | 0.72 (0.10-5.27) |  |
|  | Log-additive | --- | --- | --- | 0.87 (0.61-1.24) | 0.440 | --- | --- | 1.35 (0.83-2.17) | 0.220 |
| rs3751812 | Codominant | G/G | 239 (76.8%) | 226 (81%) | 1 | 0.450 | 157 (84%) | 171 (78.8%) | 1 | 0.400 |
|  |  | G/T | 68 (21.9%) | 48 (17.2%) | 0.77 (0.51-1.17) |  | 28 (15%) | 44 (20.3%) | 1.42 (0.84-2.41) |  |
|  |  | T/T | 4 (1.3%) | 5 (1.8%) | 1.18 (0.31-4.52) |  | 2 (1.1%) | 2 (0.9%) | 0.77 (0.11-5.66) |  |
|  | Dominant | G/G | 239 (76.8%) | 226 (81%) | 1 | 0.270 | 157 (84%) | 171 (78.8%) | 1 | 0.220 |
|  |  | G/T-T/T | 72 (23.1%) | 53 (19%) | 0.80 (0.53-1.19) |  | 30 (16%) | 46 (21.2%) | 1.38 (0.82-2.31) |  |
|  | Recessive | G/G-G/T | 307 (98.7%) | 274 (98.2%) | 1 | 0.750 | 185 (98.9%) | 215 (99.1%) | 1 | 0.750 |
|  |  | T/T | 4 (1.3%) | 5 (1.8%) | 1.24 (0.32-4.74) |  | 2 (1.1%) | 2 (0.9%) | 0.72 (0.10-5.30) |  |
|  | Log-additive | --- | --- | --- | 0.85 (0.59-1.21) | 0.360 | --- | --- | 1.29 (0.80-2.09) | 0.290 |
| **Smoking** |  | **Smokers** | | | | | **Non-smokers** | | | |
| rs9930333 | Codominant | T/T | 197 (73.2%) | 159 (72.6%) | 1 | 0.460 | 155 (67.7%) | 203 (72.8%) | 1 | 0.250 |
|  |  | G/T | 67 (24.9%) | 52 (23.7%) | 0.98 (0.64-1.49) |  | 69 (30.1%) | 67 (24%) | 0.74 (0.49-1.10) |  |
|  |  | G/G | 5 (1.9%) | 8 (3.6%) | 2.03 (0.65-6.36) |  | 5 (2.2%) | 9 (3.2%) | 1.40 (0.45-4.31) |  |
|  | Dominant | T/T | 197 (73.2%) | 159 (72.6%) | 1 | 0.810 | 155 (67.7%) | 203 (72.8%) | 1 | 0.210 |
|  |  | G/T-G/G | 72 (26.8%) | 60 (27.4%) | 1.05 (0.70-1.58) |  | 74 (32.3%) | 76 (27.2%) | 0.78 (0.53-1.15) |  |
|  | Recessive | T/T-G/T | 264 (98.1%) | 211 (96.3%) | 1 | 0.210 | 224 (97.8%) | 270 (96.8%) | 1 | 0.460 |
|  |  | G/G | 5 (1.9%) | 8 (3.6%) | 2.04 (0.65-6.37) |  | 5 (2.2%) | 9 (3.2%) | 1.52 (0.50-4.66) |  |
|  | Log-additive | --- | --- | --- | 1.11 (0.78-1.58) | 0.550 | --- | --- | 0.86 (0.62-1.21) | 0.390 |
| rs11642015 | Codominant | C/C | 218 (81%) | 172 (78.5%) | 1 | 0.280 | 179 (78.2%) | 225 (80.7%) | 1 | 0.190 |
|  |  | T/C | 47 (17.5%) | 46 (21%) | 1.28 (0.81-2.02) |  | 48 (21%) | 47 (16.9%) | 0.79 (0.50-1.24) |  |
|  |  | T/T | 4 (1.5%) | 1 (0.5%) | 0.31 (0.03-2.80) |  | 2 (0.9%) | 7 (2.5%) | 2.95 (0.60-14.63) |  |
|  | Dominant | C/C | 218 (81%) | 172 (78.5%) | 1 | 0.430 | 179 (78.2%) | 225 (80.7%) | 1 | 0.540 |
|  |  | T/C-T/T | 51 (19%) | 47 (21.5%) | 1.20 (0.76-1.88) |  | 50 (21.8%) | 54 (19.4%) | 0.87 (0.56-1.35) |  |
|  | Recessive | C/C-T/C | 265 (98.5%) | 218 (99.5%) | 1 | 0.230 | 227 (99.1%) | 272 (97.5%) | 1 | 0.130 |
|  |  | T/T | 4 (1.5%) | 1 (0.5%) | 0.29 (0.03-2.67) |  | 2 (0.9%) | 7 (2.5%) | 3.09 (0.63-15.25) |  |
|  | Log-additive | --- | --- | --- | 1.10 (0.73-1.67) | 0.640 | --- | --- | 0.97 (0.66-1.44) | 0.900 |
| rs1558902 | Codominant | T/T | 218 (81%) | 172 (78.5%) | 1 | 0.280 | 180 (78.6%) | 228 (81.7%) | 1 | 0.230 |
|  |  | A/T | 47 (17.5%) | 46 (21%) | 1.28 (0.81-2.02) |  | 47 (20.5%) | 45 (16.1%) | 0.75 (0.47-1.19) |  |
|  |  | A/A | 4 (1.5%) | 1 (0.5%) | 0.31 (0.03-2.80) |  | 2 (0.9%) | 6 (2.1%) | 2.46 (0.48-12.54) |  |
|  | Dominant | T/T | 218 (81%) | 172 (78.5%) | 1 | 0.430 | 180 (78.6%) | 228 (81.7%) | 1 | 0.380 |
|  |  | A/T-A/A | 51 (19%) | 47 (21.5%) | 1.20 (0.77-1.88) |  | 49 (21.4%) | 51 (18.3%) | 0.82 (0.53-1.28) |  |
|  | Recessive | T/T-A/T | 265 (98.5%) | 218 (99.5%) | 1 | 0.230 | 227 (99.1%) | 273 (97.8%) | 1 | 0.220 |
|  |  | A/A | 4 (1.5%) | 1 (0.5%) | 0.29 (0.03-2.67) |  | 2 (0.9%) | 6 (2.1%) | 2.59 (0.51-13.16) |  |
|  | Log-additive | --- | --- | --- | 1.10 (0.73-1.67) | 0.640 | --- | --- | 0.91 (0.61-1.36) | 0.650 |
| rs56094641 | Codominant | A/A | 217 (80.7%) | 172 (78.5%) | 1 | 0.310 | 179 (78.2%) | 224 (80.3%) | 1 | 0.200 |
|  |  | G/A | 48 (17.8%) | 46 (21%) | 1.25 (0.79-1.97) |  | 48 (21%) | 48 (17.2%) | 0.80 (0.51-1.26) |  |
|  |  | G/G | 4 (1.5%) | 1 (0.5%) | 0.31 (0.03-2.79) |  | 2 (0.9%) | 7 (2.5%) | 2.96 (0.60-14.68) |  |
|  | Dominant | A/A | 217 (80.7%) | 172 (78.5%) | 1 | 0.490 | 179 (78.2%) | 224 (80.3%) | 1 | 0.580 |
|  |  | G/A-G/G | 52 (19.3%) | 47 (21.5%) | 1.17 (0.75-1.83) |  | 50 (21.8%) | 55 (19.7%) | 0.89 (0.57-1.37) |  |
|  | Recessive | A/A-G/A | 265 (98.5%) | 218 (99.5%) | 1 | 0.230 | 227 (99.1%) | 272 (97.5%) | 1 | 0.130 |
|  |  | G/G | 4 (1.5%) | 1 (0.5%) | 0.29 (0.03-2.67) |  | 2 (0.9%) | 7 (2.5%) | 3.09 (0.63-15.25) |  |
|  | Log-additive | --- | --- | --- | 1.08 (0.71-1.64) | 0.710 | --- | --- | 0.99 (0.67-1.45) | 0.950 |
| rs3751812 | Codominant | G/G | 217 (80.7%) | 171 (78.1%) | 1 | 0.270 | 179 (78.2%) | 226 (81.6%) | 1 | 0.220 |
|  |  | G/T | 48 (17.8%) | 47 (21.5%) | 1.29 (0.82-2.02) |  | 48 (21%) | 45 (16.2%) | 0.75 (0.47-1.18) |  |
|  |  | T/T | 4 (1.5%) | 1 (0.5%) | 0.31 (0.03-2.81) |  | 2 (0.9%) | 6 (2.2%) | 2.47 (0.48-12.60) |  |
|  | Dominant | G/G | 217 (80.7%) | 171 (78.1%) | 1 | 0.410 | 179 (78.2%) | 226 (81.6%) | 1 | 0.370 |
|  |  | G/T-T/T | 52 (19.3%) | 48 (21.9%) | 1.21 (0.77-1.88) |  | 50 (21.8%) | 51 (18.4%) | 0.82 (0.52-1.27) |  |
|  | Recessive | G/G-G/T | 265 (98.5%) | 218 (99.5%) | 1 | 0.230 | 227 (99.1%) | 271 (97.8%) | 1 | 0.220 |
|  |  | T/T | 4 (1.5%) | 1 (0.5%) | 0.29 (0.03-2.67) |  | 2 (0.9%) | 6 (2.2%) | 2.60 (0.51-13.25) |  |
|  | Log-additive | --- | --- | --- | 1.11 (0.74-1.68) | 0.620 | --- | --- | 0.91 (0.61-1.35) | 0.630 |
| **Drinking** |  | **Drinkers** | | | | | **Non-drinkers** | | | |
| rs9930333 | Codominant | T/T | 185 (72.3%) | 186 (70.2%) | 1 | 0.430 | 167 (69%) | 176 (75.5%) | 1 | 0.170 |
|  |  | G/T | 66 (25.8%) | 69 (26%) | 1.02 (0.68-1.52) |  | 70 (28.9%) | 50 (21.5%) | 0.68 (0.44-1.04) |  |
|  |  | G/G | 5 (2%) | 10 (3.8%) | 2.05 (0.67-6.26) |  | 5 (2.1%) | 7 (3%) | 1.26 (0.39-4.09) |  |
|  | Dominant | T/T | 185 (72.3%) | 186 (70.2%) | 1 | 0.670 | 167 (69%) | 176 (75.5%) | 1 | 0.110 |
|  |  | G/T-G/G | 71 (27.7%) | 79 (29.8%) | 1.09 (0.74-1.61) |  | 75 (31%) | 57 (24.5%) | 0.72 (0.48-1.08) |  |
|  | Recessive | T/T-G/T | 251 (98%) | 255 (96.2%) | 1 | 0.20 | 237 (97.9%) | 226 (97%) | 1 | 0.570 |
|  |  | G/G | 5 (2%) | 10 (3.8%) | 2.04 (0.67-6.20) |  | 5 (2.1%) | 7 (3%) | 1.40 (0.43-4.51) |  |
|  | Log-additive | --- | --- | --- | 1.14 (0.81-1.60) | 0.440 | --- | --- | 0.80 (0.56-1.15) | 0.220 |
| rs11642015 | Codominant | C/C | 207 (80.9%) | 206 (77.7%) | 1 | 0.600 | 190 (78.5%) | 191 (82%) | 1 | 0.450 |
|  |  | T/C | 46 (18%) | 56 (21.1%) | 1.26 (0.80-1.96) |  | 49 (20.2%) | 37 (15.9%) | 0.77 (0.48-1.24) |  |
|  |  | T/T | 3 (1.2%) | 3 (1.1%) | 0.90 (0.17-4.73) |  | 3 (1.2%) | 5 (2.1%) | 1.51 (0.35-6.49) |  |
|  | Dominant | C/C | 207 (80.9%) | 206 (77.7%) | 1 | 0.340 | 190 (78.5%) | 191 (82%) | 1 | 0.380 |
|  |  | T/C-T/T | 49 (19.1%) | 59 (22.3%) | 1.23 (0.80-1.91) |  | 52 (21.5%) | 42 (18%) | 0.81 (0.52-1.29) |  |
|  | Recessive | C/C-T/C | 253 (98.8%) | 262 (98.9%) | 1 | 0.860 | 239 (98.8%) | 228 (97.8%) | 1 | 0.530 |
|  |  | T/T | 3 (1.2%) | 3 (1.1%) | 0.87 (0.17-4.52) |  | 3 (1.2%) | 5 (2.1%) | 1.59 (0.37-6.79) |  |
|  | Log-additive | --- | --- | --- | 1.18 (0.79-1.77) | 0.410 | --- | --- | 0.88 (0.59-1.32) | 0.550 |
| rs1558902 | Codominant | T/T | 207 (80.9%) | 207 (78.1%) | 1 | 0.670 | 191 (78.9%) | 193 (82.8%) | 1 | 0.500 |
|  |  | A/T | 46 (18%) | 55 (20.8%) | 1.22 (0.78-1.91) |  | 48 (19.8%) | 36 (15.4%) | 0.76 (0.47-1.22) |  |
|  |  | A/A | 3 (1.2%) | 3 (1.1%) | 0.90 (0.17-4.70) |  | 3 (1.2%) | 4 (1.7%) | 1.20 (0.26-5.52) |  |
|  | Dominant | T/T | 207 (80.9%) | 207 (78.1%) | 1 | 0.420 | 191 (78.9%) | 193 (82.8%) | 1 | 0.310 |
|  |  | A/T-A/A | 49 (19.1%) | 58 (21.9%) | 1.20 (0.77-1.86) |  | 51 (21.1%) | 40 (17.2%) | 0.79 (0.49-1.25) |  |
|  | Recessive | T/T-A/T | 253 (98.8%) | 262 (98.9%) | 1 | 0.860 | 239 (98.8%) | 229 (98.3%) | 1 | 0.760 |
|  |  | A/A | 3 (1.2%) | 3 (1.1%) | 0.87 (0.17-4.52) |  | 3 (1.2%) | 4 (1.7%) | 1.26 (0.28-5.78) |  |
|  | Log-additive | --- | --- | --- | 1.16 (0.77-1.73) | 0.480 | --- | --- | 0.84 (0.55-1.27) | 0.410 |
| rs56094641 | Codominant | A/A | 206 (80.5%) | 205 (77.4%) | 1 | 0.600 | 190 (78.5%) | 191 (82%) | 1 | 0.460 |
|  |  | G/A | 47 (18.4%) | 57 (21.5%) | 1.25 (0.80-1.95) |  | 49 (20.2%) | 37 (15.9%) | 0.77 (0.48-1.24) |  |
|  |  | G/G | 3 (1.2%) | 3 (1.1%) | 0.91 (0.17-4.73) |  | 3 (1.2%) | 5 (2.1%) | 1.51 (0.35-6.50) |  |
|  | Dominant | A/A | 206 (80.5%) | 205 (77.4%) | 1 | 0.350 | 190 (78.5%) | 191 (82%) | 1 | 0.380 |
|  |  | G/A-G/G | 50 (19.5%) | 60 (22.6%) | 1.23 (0.80-1.90) |  | 52 (21.5%) | 42 (18%) | 0.82 (0.52-1.29) |  |
|  | Recessive | A/A-G/A | 253 (98.8%) | 262 (98.9%) | 1 | 0.860 | 239 (98.8%) | 228 (97.8%) | 1 | 0.530 |
|  |  | G/G | 3 (1.2%) | 3 (1.1%) | 0.87 (0.17-4.52) |  | 3 (1.2%) | 5 (2.1%) | 1.59 (0.37-6.79) |  |
|  | Log-additive | --- | --- | --- | 1.18 (0.79-1.77) | 0.410 | --- | --- | 0.88 (0.59-1.32) | 0.550 |
| rs3751812 | Codominant | G/G | 206 (80.5%) | 206 (78%) | 1 | 0.680 | 190 (78.5%) | 191 (82.3%) | 1 | 0.520 |
|  |  | G/T | 47 (18.4%) | 55 (20.8%) | 1.22 (0.78-1.90) |  | 49 (20.2%) | 37 (15.9%) | 0.77 (0.48-1.23) |  |
|  |  | T/T | 3 (1.2%) | 3 (1.1%) | 0.90 (0.17-4.71) |  | 3 (1.2%) | 4 (1.7%) | 1.21 (0.27-5.57) |  |
|  | Dominant | G/G | 206 (80.5%) | 206 (78%) | 1 | 0.420 | 190 (78.5%) | 191 (82.3%) | 1 | 0.330 |
|  |  | G/T-T/T | 50 (19.5%) | 58 (22%) | 1.20 (0.77-1.85) |  | 52 (21.5%) | 41 (17.7%) | 0.79 (0.50-1.26) |  |
|  | Recessive | G/G-G/T | 253 (98.8%) | 261 (98.9%) | 1 | 0.870 | 239 (98.8%) | 228 (98.3%) | 1 | 0.750 |
|  |  | T/T | 3 (1.2%) | 3 (1.1%) | 0.87 (0.17-4.53) |  | 3 (1.2%) | 4 (1.7%) | 1.27 (0.28-5.83) |  |
|  | Log-additive | --- | --- | --- | 1.15 (0.77-1.72) | 0.480 | --- | --- | 0.85 (0.56-1.28) | 0.430 |

ONFH: osteonecrosis of the femoral head; SNP: single nucleotide polymorphism; OR: odds ratio; 95% CI: 95% confidence interval.

*p* values were calculated using logistic regression analysis adjusted by gender, age, smoking and/or drinking.

**Suppl_Table 3.** Association between ***FTO*** polymorphisms and **ONFH risk** in the stratified analysis by clinical stages

| **SNP ID** | **Model** | **Genotype** | **III/IV patients** | **I/II patients** | **OR (95% CI)** | **P-value** |
| --- | --- | --- | --- | --- | --- | --- |
| rs9930333 | Codominant | T/T | 51 (76.1%) | 138 (71.9%) | 1 | 0.890 |
|  |  | G/T | 13 (19.4%) | 47 (24.5%) | 1.19 (0.57-2.47) |  |
|  |  | G/G | 3 (4.5%) | 7 (3.6%) | 1.00 (0.23-4.37) |  |
|  | Dominant | T/T | 51 (76.1%) | 138 (71.9%) | 1 | 0.670 |
|  |  | G/T-G/G | 16 (23.9%) | 54 (28.1%) | 1.16 (0.59-2.28) |  |
|  | Recessive | T/T-G/T | 64 (95.5%) | 185 (96.3%) | 1 | 0.960 |
|  |  | G/G | 3 (4.5%) | 7 (3.6%) | 0.97 (0.22-4.17) |  |
|  | Log-additive | --- | --- | --- | 1.10 (0.63-1.90) | 0.740 |
| rs11642015 | Codominant | C/C | 55 (82.1%) | 149 (77.6%) | 1 | 0.810 |
|  |  | T/C | 11 (16.4%) | 41 (21.4%) | 1.24 (0.57-2.68) |  |
|  |  | T/T | 1 (1.5%) | 2 (1%) | 1.59 (0.13-19.63) |  |
|  | Dominant | C/C | 55 (82.1%) | 149 (77.6%) | 1 | 0.540 |
|  |  | T/C-T/T | 12 (17.9%) | 43 (22.4%) | 1.26 (0.60-2.66) |  |
|  | Recessive | C/C-T/C | 66 (98.5%) | 190 (99%) | 1 | 0.730 |
|  |  | T/T | 1 (1.5%) | 2 (1%) | 1.54 (0.12-18.94) |  |
|  | Log-additive | --- | --- | --- | 1.24 (0.64-2.43) | 0.520 |
| rs1558902 | Codominant | T/T | 55 (82.1%) | 151 (78.7%) | 1 | 0.930 |
|  |  | A/T | 11 (16.4%) | 39 (20.3%) | 1.07 (0.49-2.33) |  |
|  |  | A/A | 1 (1.5%) | 2 (1%) | 1.55 (0.13-19.15) |  |
|  | Dominant | T/T | 55 (82.1%) | 151 (78.7%) | 1 | 0.800 |
|  |  | A/T-A/A | 12 (17.9%) | 41 (21.4%) | 1.10 (0.52-2.34) |  |
|  | Recessive | T/T-A/T | 66 (98.5%) | 190 (99%) | 1 | 0.730 |
|  |  | A/A | 1 (1.5%) | 2 (1%) | 1.54 (0.12-18.94) |  |
|  | Log-additive | --- | --- | --- | 1.12 (0.57-2.19) | 0.750 |
| rs56094641 | Codominant | A/A | 55 (82.1%) | 149 (77.6%) | 1 | 0.850 |
|  |  | G/A | 11 (16.4%) | 41 (21.4%) | 1.20 (0.55-2.59) |  |
|  |  | G/G | 1 (1.5%) | 2 (1%) | 1.58 (0.13-19.49) |  |
|  | Dominant | A/A | 55 (82.1%) | 149 (77.6%) | 1 | 0.590 |
|  |  | G/A-G/G | 12 (17.9%) | 43 (22.4%) | 1.22 (0.58-2.58) |  |
|  | Recessive | A/A-G/A | 66 (98.5%) | 190 (99%) | 1 | 0.730 |
|  |  | G/G | 1 (1.5%) | 2 (1%) | 1.54 (0.12-18.94) |  |
|  | Log-additive | --- | --- | --- | 1.21 (0.62-2.37) | 0.570 |
| rs3751812 | Codominant | G/G | 55 (82.1%) | 149 (77.6%) | 1 | 0.860 |
|  |  | G/T | 11 (16.4%) | 41 (21.4%) | 1.18 (0.54-2.56) |  |
|  |  | T/T | 1 (1.5%) | 2 (1%) | 1.57 (0.13-19.43) |  |
|  | Dominant | G/G | 55 (82.1%) | 149 (77.6%) | 1 | 0.620 |
|  |  | G/T-T/T | 12 (17.9%) | 43 (22.4%) | 1.21 (0.57-2.55) |  |
|  | Recessive | G/G-G/T | 66 (98.5%) | 190 (99%) | 1 | 0.730 |
|  |  | T/T | 1 (1.5%) | 2 (1%) | 1.54 (0.12-18.94) |  |
|  | Log-additive | --- | --- | --- | 1.20 (0.61-2.34) | 0.590 |
| rs62033406 | Codominant | A/A | 15 (22.4%) | 47 (24.6%) | 1 | 0.440 |
|  |  | G/A | 39 (58.2%) | 98 (51.3%) | 0.70 (0.34-1.46) |  |
|  |  | G/G | 13 (19.4%) | 46 (24.1%) | 1.08 (0.44-2.64) |  |
|  | Dominant | A/A | 15 (22.4%) | 47 (24.6%) | 1 | 0.520 |
|  |  | G/A-G/G | 52 (77.6%) | 144 (75.4%) | 0.80 (0.39-1.61) |  |
|  | Recessive | A/A-G/A | 54 (80.6%) | 145 (75.9%) | 1 | 0.390 |
|  |  | G/G | 13 (19.4%) | 46 (24.1%) | 1.37 (0.66-2.86) |  |
|  | Log-additive | --- | --- | --- | 1.02 (0.67-1.57) | 0.910 |

ONFH: osteonecrosis of the femoral head; SNP: single nucleotide polymorphism; OR: odds ratio; 95% CI: 95% confidence interval.

*p* values were calculated using logistic regression analysis adjusted by gender, age, smoking and/or drinking.
